# Evaluating Novel Quantification Methods for Infectious Baculoviruses

**DOI:** 10.3390/v15040998

**Published:** 2023-04-19

**Authors:** Keven Lothert, Elena Bagrin, Michael W. Wolff

**Affiliations:** Institute of Bioprocess Engineering and Pharmaceutical Technology, Department Life Science Engineering, University of Applied Sciences Mittelhessen (THM), 35390 Giessen, Germany; keven.lothert@lse.thm.de (K.L.);

**Keywords:** flow cytometry, infectivity titer, virus quantification, process monitoring, native baculovirus, quantitative polymerase chain reaction, viral RNA, gp64 protein

## Abstract

Accurate and rapid quantification of (infectious) virus titers is of paramount importance in the manufacture of viral vectors and vaccines. Reliable quantification data allow efficient process development at a laboratory scale and thorough process monitoring in later production. However, current gold standard applications, such as endpoint dilution assays, are cumbersome and do not provide true process analytical monitoring. Accordingly, flow cytometry and quantitative polymerase chain reaction have attracted increasing interest in recent years, offering various advantages for rapid quantification. Here, we compared different approaches for the assessment of infectious viruses, using a model baculovirus. Firstly, infectivity was estimated by the quantification of viral nucleic acids in infected cells, and secondly, different flow cytometric approaches were investigated regarding analysis times and calibration ranges. The flow cytometry technique included a quantification based on post-infection fluorophore expression and labeling of a viral surface protein using fluorescent antibodies. Additionally, the possibility of viral (m)RNA labeling in infected cells was investigated as a proof of concept. The results confirmed that infectivity assessment based on qPCR is not trivial and requires sophisticated method optimization, whereas staining of viral surface proteins is a fast and feasible approach for enveloped viruses. Finally, labeling of viral (m)RNA in infected cells appears to be a promising opportunity but will require further research.

## 1. Introduction

Viruses play an important role in many pharmaceutical applications to achieve immune responses [1,2], deliver genetic information [3,4], or act as oncolytic agents [5,6]. Viruses used as human therapeutics are usually produced in vitro, using cell culture or egg-based production systems [7,8,9]. In addition, after product amplification, a rigorous purification process is required to allow safe and efficient administration to the patient [10]. During all steps of this production, proper process monitoring is desirable, e.g., to enable the surveillance of the product titers during cultivation or to estimate the recovery yield of individual purification steps. Analytical techniques for virus quantification can be cumbersome and time consuming and are highly dependent on the composition of the target product and on the information required [11]. For the quantification of infectious viruses, i.e., those used as live (attenuated) vaccines, the mere determination of the total particle concentration [12] or the amount of viral nucleic acids in a sample [13,14] is usually not sufficient. However, several reports have described the quantification of infectious virus particles using digital or quantitative polymerase chain reaction (dPCR or qPCR, respectively) [15,16,17]. Specific nucleic acid sequences have been detected, allowing conclusions on the integrity of the viral genome. From this, correlations and predictions of the actual infectivity can be made. Using these techniques, it is possible to estimate the concentration of infectious viruses in a sample within a few hours. However, PCR methods usually involve high costs per analysis and the infectious titer is derived indirectly. Therefore, designated infectivity assays are usually required. The most common approaches are the fifty-percent tissue culture infectious dose (TCID_50_) assay [18,19,20] and the plaque forming unit (PFU) assay [21,22]. Both assays rely on the infection of serial virus dilutions, i.e., they are endpoint dilution assays, and the readout is based on cytopathic effects. Although detection can be facilitated by the use of (fluorescent) dyes, several virus replication cycles are generally required before notable effects are visible. This results in a long analysis time of up to two weeks, depending on the cell type and virus used. Since the actual virus production process, including upstream and downstream processing, is usually completed within a week, these traditional assays do not allow process monitoring but merely a retrospective batch evaluation. In recent years, flow-cytometry-based infectivity assays have gained increasing interest [11,23,24]. In this approach, susceptible host cells are infected with the virus, and the readout is performed according to fluorophore expression in infected cells or via the labeling of expressed virus proteins. Due to the increased sensitivity of the flow cytometric titration and to avoid secondary infection cycles, the readout is usually performed after one replication cycle within 7–48 h.

In this study, different approaches for the quantification of (infectious) viruses were compared to evaluate the analysis times, linear range, and reproducibility of the different techniques. In order to potentially derive platform approaches for a variety of viruses, the evaluations were carried out using baculovirus (BV) as a model. Insect pathogenic BVs in the context of the baculovirus expression vector system (BEVS) are well known since the 1980s as an expression system for a variety of pharmaceutical proteins, including structural proteins for the assembly of virus-like particles (VLPs) [25,26]. Furthermore, BV has been described as a potential tool for gene therapy applications [27]. As a result, a variety of publications have focused on the production [28,29,30,31,32] and purification [33,34,35] of the BV, as well as its quantification [32,36]. The latter has mainly been performed using endpoint dilution assays, plaque assays, and qPCR. In addition, a flow cytometric assay has been described to quantify the BVs based on staining of the viral DNA inside the virus [37] as well as using the labeling of the viral gp64 surface protein [38].

In summary, we performed qPCR evaluations of total viral nucleic acid copies in free virus as well as in infected cells. We also evaluated different flow-cytometry-based quantification methods with respect to the analysis time, the sensitivity, and the calibration range. Targets included the direct detection of fluorophore expression, the labeling of viral envelope proteins found on the surface of infected cells, and the viral mRNA translated inside the cells. The latter was achieved by combining labeling by the hybridization of a probe and fluorophores to the RNA with subsequent flow cytometric detection and was carried out as a proof of concept only. For all approaches, infection kinetics were determined to identify the earliest possible readout that would allow reproducible analysis. By using a model BV, this study aims to demonstrate a universal method that allows rapid and accurate determination of infectious virus titers, which can be easily transferred to other viruses, irrespective of the virus composition (with or without envelope), the nucleic acid content (DNA or RNA), and the presence or absence of a reporter fluorophore. To the best of our knowledge, the possibility of quantification by flow cytometry after staining of viral nucleic acids in infected cells has not yet been investigated and reported elsewhere. Furthermore, no comparative evaluation of the different techniques has been published to date.

## 2. Materials and Methods

### 2.1. Generation of Baculovirus Stock

Two different BV genotypes, with and without green fluorescent protein expression (BV-GFP and BV-wt, respectively), were generated using pFastBac vectors, from the Viral Core Facility of the University Hospital Hamburg-Eppendorf, Germany, kindly provided by Dr. Ingke Braren. BVs from the first population (P1) were amplified in *Spodoptera frugiperda* (Sf9) cells cultivated in serum-free medium (Sf900 II, Gibco, Life Technologies GmbH, Darmstadt, Germany). The total amount of virus was further increased by subsequent rounds of amplification up to the third BV passage (P3). For each round of amplification, 1 × 10^6^ cells mL^−1^ were seeded in suspension shake flasks and infected with the respective BV at a multiplicity of infection (MOI) of 0.02. The virus cultivation was stopped after about 4 days when cell viability fell below 50%. Upon harvest, the suspension was clarified by sequential centrifugation (500× *g*, 5 min; 3000× *g*, 10 min; 4600× *g*, 20 min). The clarified supernatants were pooled and stored at 4 °C (up to 2 months) or at −80 °C (long-term storage).

If necessary, the BV stock was further concentrated by tangential flow filtration. Depending on the scale, either an Amicon^®^ stirred cell with 300 kDa polyether sulfone filters (up to 200 mL starting volume, Merck KGaA, Darmstadt, Germany) or the Sartoflow^®^ Smart system with 300 kDa Hydrosart^®^ filters (up to 1 L starting volume, Sartorius Stedim Biotech GmbH, Göttingen, Germany) was used.

### 2.2. Quantification of Stock Titer

To estimate the amount of infectious virus titers in the clarified and concentrated stock for the evaluation of subsequent assays, a PFU assay was applied in a 6-well format as described elsewhere [28]. A 2% (*w*/*v*) agarose overlay was used during the incubation and for the readout, the wells were stained with Neutral Red to identify the plaques containing the dead cells. The resulting BV titers were confirmed by a classical TCID_50_ assay as described by Merkling et al. [39].

For a better understanding, all values describing infectious virus titers are displayed as infectious units (IU)/mL and are correlated to the standard calibration sample, adjusted according to the TCID_50_/mL value.

### 2.3. Quantitative Polymerase Chain Reaction

The qPCR experiments were performed as previously described for another baculovirus by our group with minor adaptations [33]. Briefly, viral DNA was extracted using the PureLink™ Viral RNA/DNA Mini Kit (Life Technologies GmbH, Darmstadt, Germany), according to the manufacturers’ instructions. For the actual qPCR, the QuantiNova™ SYBR^®^ Green PCR Kit (Qiagen, Hilden, Germany) was applied. Preceding the experiments, optimum forward and reversed primers targeting the f1 ori of the shuttle plasmid, suitable for both virus genotypes, were identified (f: ^5^‘CCGCTCCTTTCGCTTTCTTCC‘^3^ r: ^5^‘GCCCACTACGTGAACCATCACC‘^3^, obtained from Sigma Aldrich, Merck KGaA). For each measurement, 4 µL of the DNA extract was used. The amplification was performed using 96-well plates and a light cycler (AriaMx, Agilent, Santa Clara, CA, USA). The qPCR program comprised 40 cycles of denaturation (15 s, 95 °C), annealing (10 s, 60 °C), and elongation (10 s, 72 °C). In the end, a melting curve analysis (72–90 °C within 20 min) was conducted to confirm specificity of the amplificates.

To identify the time of virus replication and derive conclusions on the virus infectivity based on qPCR, cells were infected with different virus dilutions. The incubation was stopped at various time points between 5 min and 18 h by centrifugation at 500× *g* for 5 min. Afterward, the cell pellet was washed with phosphate-buffered saline (PBS), and the DNA was extracted as described above.

### 2.4. Flow Cytometric Titration

The determination of infectious virus titers using flow cytometry was carried out either on the basis of (i) direct detection of the virus-related GFP expression in infected cells, (ii) labeling of the viral envelope glycoprotein gp64, or (iii) staining of viral nucleic acids in infected cells by the hybridization of probes. In all cases, Sf9 cells were seeded in varying cell concentrations (75,000–150,000 cells per well) in 150 µL serum-free medium (Sf900 II, Gibco) into 96-well suspension plates (round bottom, Sarstedt). Directly afterward, 50 µL of either medium blank or virus sample at different dilutions (diluted with medium from stock in Section 2.1) were added, followed by incubation at 28 °C. The incubation time was optimized for the individual target (i–iii) and was evaluated between 5 min and 18 h.

Following the incubation, the plate was centrifuged (500× *g*, 3 min), the supernatant discarded, and the cell pellet washed with PBS. After an additional centrifugation, the cells were fixed in a PBS buffer containing 1% paraformaldehyde (*v*/*v*), 2 mM EDTA, and 2% FCS (*v*/*v*).

To estimate infectivity based on GFP expression (i), the percentage of fluorescent (i.e., infected) cells was analyzed using a flow cytometer (Guava^®^ easyCyte 12 HT, Luminex, Austin, TX US) at a fluorescence excitation and emission of 488 and 525 nm, respectively.

For (ii), the washed cells were resuspended in a fixation buffer containing either a phycoerythrin- (PE) or an allophycocyanin (APC)-labeled anti-gp64 antibody (ThermoFisher Scientific, Waltham, MA USA). The antibody concentration and the duration of the incubation were individually optimized, and the optimum conditions are stated in Table 1. After incubation with the fluorescent antibody at 4 °C, 150 µL of fixation buffer was added to each well; the plate was centrifuged; and the pellets were re-suspended in 150 µL of the fixation buffer. Afterward, positive, i.e., infected, cells were determined using a flow cytometer (PE: yellow fluorescence, excitation: 488 nm, emission: 583 nm; APC: red fluorescence, excitation: 640 nm, emission: 661 nm), and the percentage of fluorescent cells was correlated to the virus titer used for the infection.

The background fluorescence was evaluated for all approaches using negative controls without virus infection and was subtracted from the measured results. Optimum parameters for (i) and (ii) regarding the sample preparation are depicted in Table 1.

Ultimately, as a proof of concept for the quantification based on viral RNA labeling (iii), the PrimeFlow RNA Assay kit (Thermo Fisher Scientific) was applied according to the manufacturers’ instructions. The procedure was conducted using 96-well V-bottom plates. The Sf9 cells were seeded in a volume of 100 µL at a concentration of 1.0 × 10^7^ cells/mL and infected with 100 µL virus stock at an MOI of 1. The infection kinetic was evaluated between 1 h and 4 h. After the incubation, the cells were washed, fixed, and permeabilized, and the RNA was additionally fixed using buffer solutions included in the kit. A custom-made probe targeting the mRNA of the baculoviral gp64 protein (sequence A: GAGCATTCGCCAGCAGTGCG) was added and hybridization was carried out at 40 °C for 2 h. Signal amplification was then performed using amplification buffers provided in the kit. After the completion of the protocol, infected cells were counted based on the detection of Alexa Fluor 647 (excitation: 642, emission: 661).

## 3. Results

The aim of this study was to compare different flow cytometric protocols in order to evaluate the quantification of BV as a model. To enable a thorough comparison, a qPCR method was first established as a reference to estimate the virus content in the samples and to assess the possibility of deriving infectivity results from qPCR data.

### 3.1. qPCR as a Referenrence Method for BV Quantification

**Figure 1:**
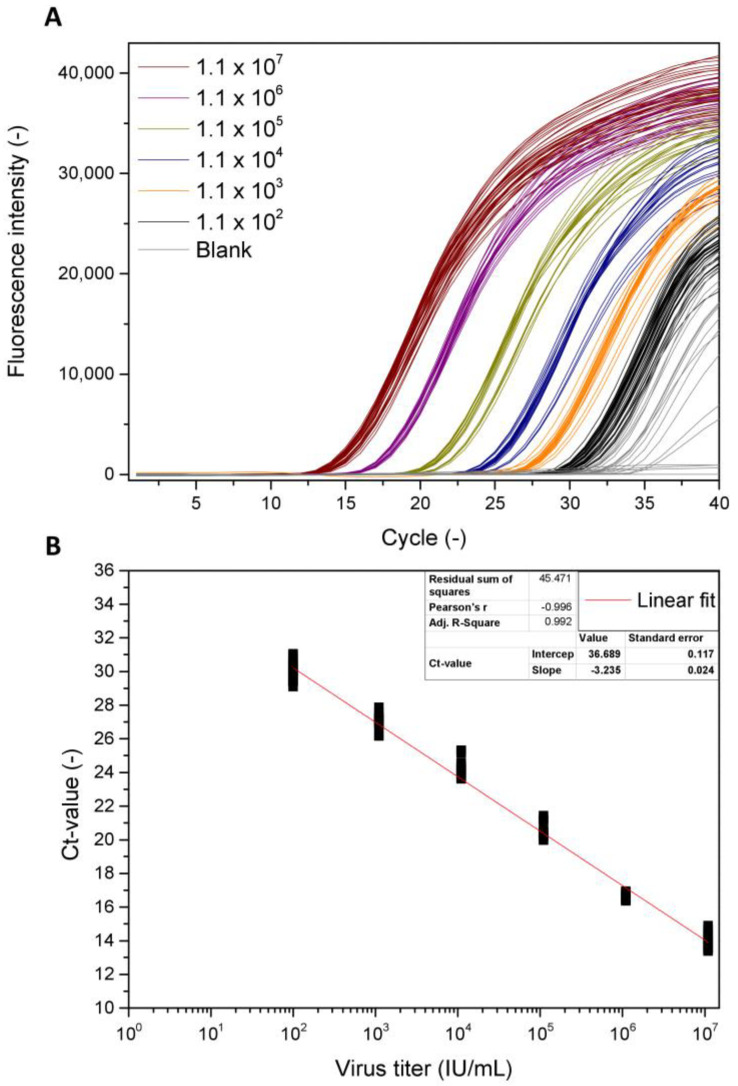
Amplification plots for the qPCR evaluation of different baculovirus concentrations in the range of 0 (i.e., blank)−1.1 × 10^7^ IU/mL (**A**) as well as the linear regression over the concentration range (**B**). The number of replicate measurements was *n* = 39 for 1.1 × 10^2^ IU/mL and 1.1 × 10^7^ IU/mL and *n* = 18 for all other concentrations and the blank.

For the application of the qPCR, in preceding evaluations, optimum forward and reversed primers were designed in order to successfully amplify the BV target DNA. The performance of the actual qPCR was later evaluated considering its reproducibility and the applicable calibration range (Figure 1A). The virus titer of the calibration stock used during these evaluations was determined by a PFU assay and confirmed by a TCID_50_ assay as indicated in Section 2.2. For convenience, all values referring to the infectious virus titer are stated as infectious units (IU)/mL. The data indicate a linear correlation between the virus titer and the measured cycle threshold over a calibration range starting from 1.1 × 10^7^ IU/mL to 1.1 × 10^2^ IU/mL at an R^2^ of 0.992 (Figure 1B). Repeated sample preparations, i.e., replicates of the DNA isolation, as well as replicates of the actual qPCR measurement, showed maximum relative deviations of the individual cycle thresholds at a concentration of about 5%. For the highest and lowest evaluated concentrations of 1.1 × 10^7^ IU/mL and 1.1 × 10^2^ IU/mL, the corresponding cycle thresholds were 13.85 ± 0.37 and 29.95 ± 0.32, respectively. Additionally, the lowest calibration sample could be clearly distinguished from the values obtained for blank samples, i.e., cell culture medium without virus, which were at about 33.11 ± 1.21. The efficiency of the qPCR procedure for the analyzed calibration samples was at about 1.04.

#### 3.1.1. Estimating the Virus Infection Kinetic Based on qPCR Measurements

**Figure 2:**
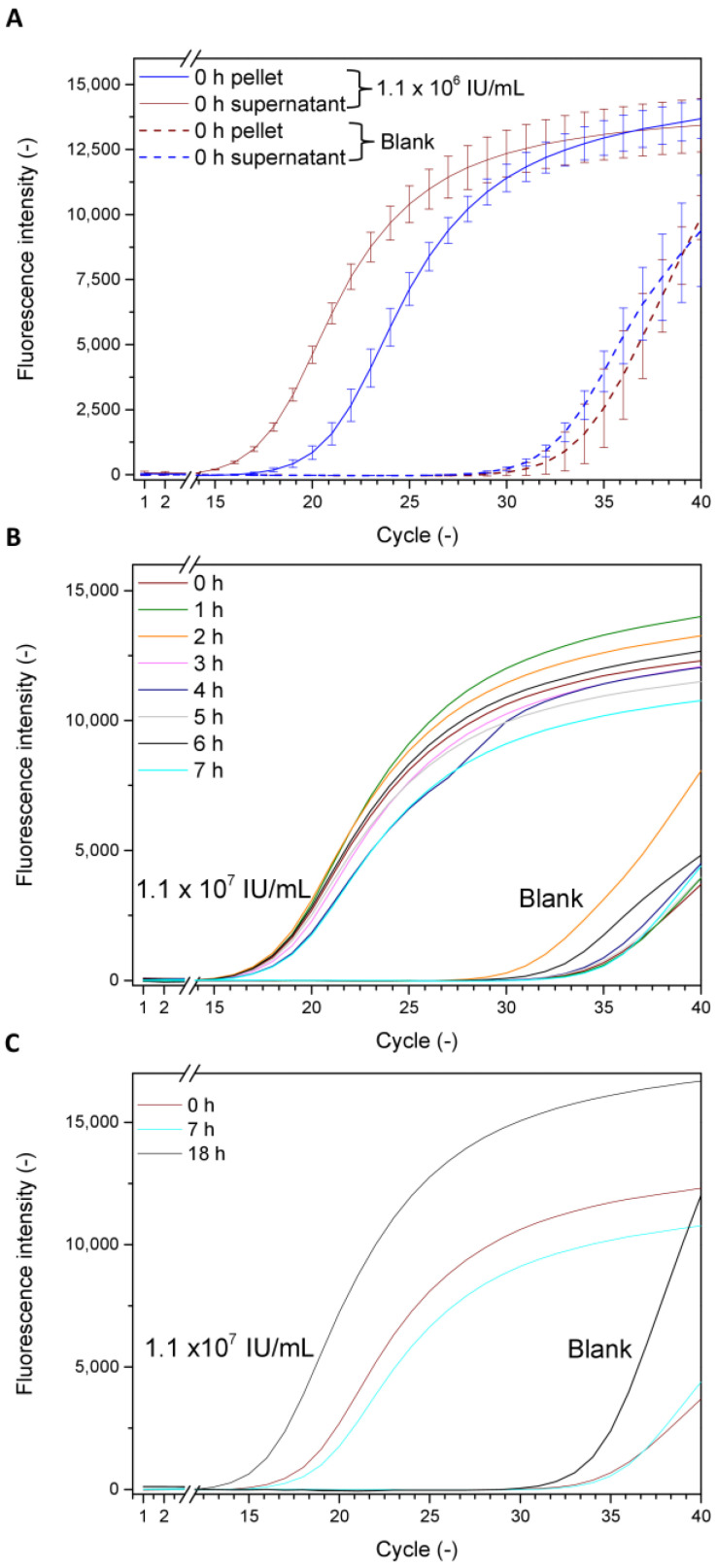
Amplification plots of the baculovirus DNA by qPCR measurements. The DNA was extracted from samples taken either from the supernatant or from the cell pellet of infected cells after 5 min (i.e., 0 h) of incubation and compared to a negative control (**A**). The amount of viral DNA detectable in the cell pellet was monitored over the course of 7 h (**B**) and compared to values after 18 h of infection (**C**). All samples were prepared in triplicates, with error bars only being displayed in (**A**) to allow a clearer view of the data.

As the qPCR measurement was set up to be a robust and reproducible reference method (see previous section), virus infection and amplification in the cells was monitored over 18 h. It was noticed that viral DNA could be detected in the cell pellet directly after 5 min of infection (Figure 2A), although the value was lower than compared to the virus remaining in the supernatant. Over the course of up to seven hours, no notable increase of the virus amount in the cell pellet was seen (Figure 2B). After an overnight incubation of cells with the virus, an increase of the viral DNA amount in the cell pellet was observed (Figure 2C). While Figure 2 depicts a comparison of the results for one particular virus concentration (1.1 × 10^6^ IU/mL) compared to the blank to allow an easier observation of the results, a thorough picture including all evaluated concentrations is shown in the supplements (Figure S1).

### 3.2. Flow Cytometric Quantification

**Figure 3:**
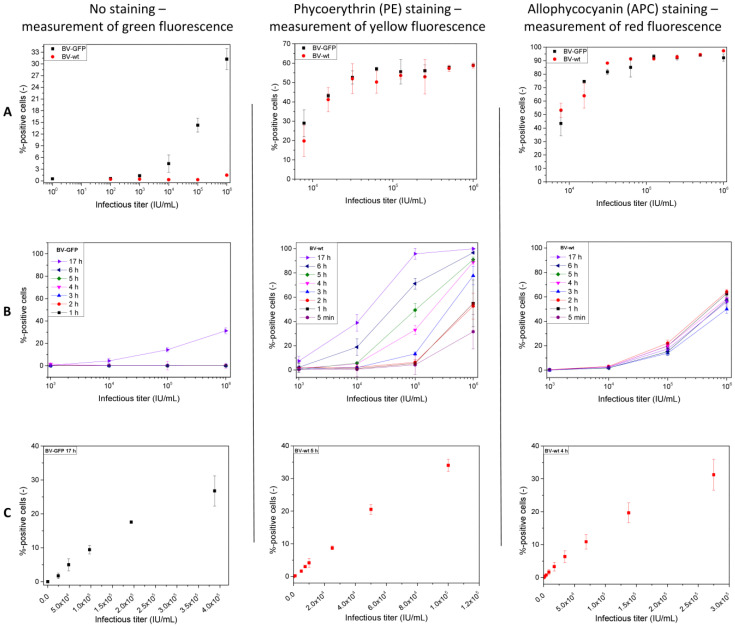
Development of the flow cytometric quantification protocol for baculoviruses (either wild-type or virus expressing the green fluorescent protein, BV-wt and BV-GFP, respectively). In a first approach, (**A**) the detection capability for both viruses was determined without further method optimization after 18 h of cell infection and subsequent flow cytometric detection of fluorescent cells. Depending on the staining procedure, green (no staining, left column), yellow (PE staining, middle column), or red fluorescence (APC staining, right column) was evaluated. The infection kinetics of the three approaches (**B**) indicate the earliest possible time of quantification at which the linear calibration range was subsequently determined for each individually optimized strategy (**C**). Error bars depict standard deviation of technical triplicates (**A**,**B**) and 18 replicates with *n* = 6 on three different days for (**C**).

For the development of the flow cytometric detection assay, initially, BV-GFP and BV-wt were used to infect Sf9 cells, and after an incubation of 18 h, the cells were evaluated for the presence of fluorescence. This was achieved either by directly measuring the green fluorescence (Figure 3A, left column) or measuring yellow and red fluorescence after labeling of the baculoviral envelope protein gp64 on the surface of infected cells by using PE- or APC-conjugated antibodies (Figure 3A, middle and right). This proof of concept shows a concentration-dependent measurement of fluorescent cells for the BV-GFP only and not for the BV-wt. On contrary, the samples that were stained with PE or APC allowed the detection of both BV types, without notable differences between them at similar virus concentrations. However, the APC-treated samples, resulted generally in higher values for the detected fluorescence, with differences of about 20–30% as compared to PE staining.

After these proof-of-concept evaluations, infection kinetics were determined for the three different detection approaches (Figure 3B). Unlabeled samples were evaluated for the BV-GFP only, whereas the BV-wt was used for the PE and APC measurements. It was observed that GFP-positive cells were measured only after an overnight incubation, in this case 17 h, whereas no fluorescence signal could be detected within the first six hours post-infection. In contrast to that, for the PE- and APC-staining procedures, positive signals were already visible after five minutes of incubation (Figure 3B, middle and right). For PE staining, the signal intensities increased in correlation with longer incubation times while, at the same time, the standard deviation of replicate measurements decreased. For APC staining, the differences between the individual incubation times were less distinct, and no notable changes were observed for an increasing number of hours. Accordingly, the optimum incubation times were set to 17 h (no staining), 5 h (PE staining), and 4 h (APC staining), respectively (Table 1). The incubation times were chosen to allow a successful quantification and to minimize the standard deviation of repeated measurements, especially considering the inter-day reproducibility.

After the evaluation of the optimum incubation time, each protocol was optimized regarding the seeding cell density, the amount of fluorescent antibody used for the staining, if applicable, and the incubation time of the latter (see Table 1, Section 2.4). Using these optimized conditions, the linear calibration range was determined for each procedure (Figure 3C). In general, a linear correlation between percentage of fluorescent cells and infectious virus titer was visible for a maximum of 25–40% fluorescent cells. The linear calibration (R^2^ > 0.99) of BV-GFP was possible for titers up to 2.5 × 10^5^ IU/mL (Figure 3C, left), whereas the upper limit of the curve was about 7.0 × 10^4^ IU/mL using PE staining and 1.4 × 10^5^ IU/mL for the APC protocol (Figure 3C, middle and right). On the contrary, the lowest possible concentration that could be distinguished from the background noise, i.e., from the negative control was 1.5 × 10^4^ IU/mL for GFP and about 1.0 × 10^3^ IU/mL for the PE and APC samples, respectively. The reproducibility of the three approaches was confirmed with triplicate measurements for each concentration on three different analysis days using fresh cells.

### 3.3. Labeling of Virus-Derived mRNA in Infected Cells for Flow Cytometric Detection

**Figure 4:**
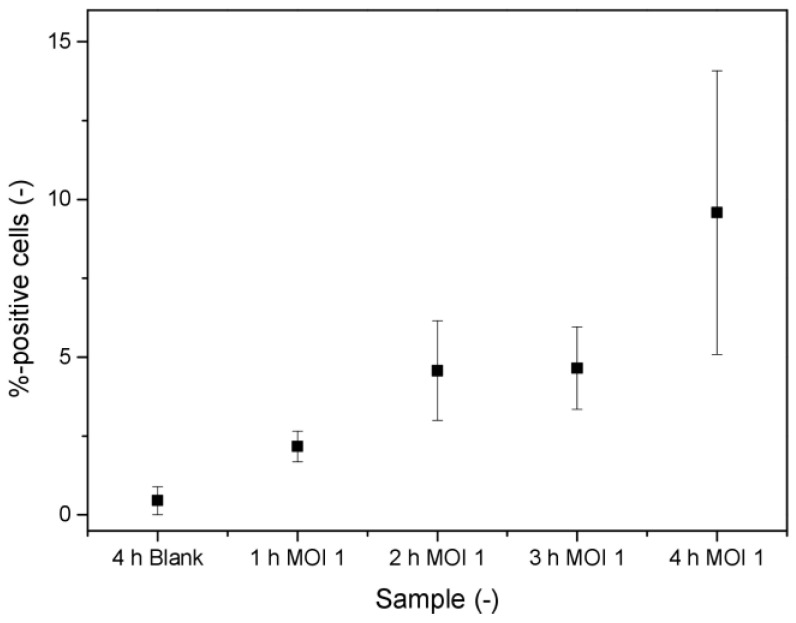
Quantification of the percentage of fluorescing cells after BV infection and fluorescent labeling of the mRNA of the viral gp64 protein within the first four hours of infection. A multiplicity of infection (MOI) of 1 was used and compared to a negative control without virus infection (error bars indicate the standard deviation of technical triplicates).

In a final approach, the flow cytometric quantification was evaluated on the basis of viral mRNA labeling in infected cells as a proof-of-concept investigation. Without further optimization, Sf9 cells were infected with BV-wt at an MOI of 1 and incubated for up to 4 h, and afterward, mRNA of the baculoviral gp64 protein was stained using a commercial kit and custom probes. It was observed that the percentage of detectable positive cells using that approach increased within the first 4 h post-infection, allowing a clear distinction from the negative control (blank) sample (Figure 4).

## 4. Discussion

### 4.1. qPCR as a Referenrence Method for BV Quantification

First, we described the setup of a classical qPCR assay for the quantification of BVs. The method was highly reproducible, taking into account inter- and intra-day replicates, and showed linearity over six log levels between 1.1 × 10^2^ and 1.1 × 10^7^ IU/mL. PCR techniques have long been applied for the quantification of viruses, employing either qPCR or, more recently, dPCR techniques [11]. For BVs, we previously described a PCR method for the monitoring of the virus levels during a purification procedure of attenuated *Autographa californica* multicapsid nucleopolyhedrovirus for gene therapy applications within a similar calibration range (1.0 × 10^3^–1.0 × 10^8^ pfu/mL) [33]. Although the technique there allowed a rapid assessment of the presence of viral genomic information in a given sample, no conclusion could be drawn from the data as to the actual infectious titer. Despite the fact that the standard stock sample used for the calibration curve was evaluated by classical TCID_50_ or plaque assays, preservation of infectivity is not guaranteed for equivalent PCR results. For other viruses, information on the viral infectivity has been estimated from qPCR data either by amplifying specific nucleic acid sequences, e.g., those present only in actively replicating viruses [40], or by correlating genomic integrity with virus activity, i.e., infectivity [16,17,41]. However, suitable target sequences can be difficult to find, and, in addition, the performance of the analytical qPCR can be affected by changes in the product composition, e.g., attenuation of the virus genotype. Therefore, in order to identify a possible platform quantification approach, we evaluated the qPCR method using the infected cells rather than the virus supernatant as a sample. The data show that the virus was detectable in the cell pellet early after the infection (see Section 3.1.1). However, this does not allow conclusions to be drawn about the infectivity of the virus, as viral DNA is detected regardless of whether the virus has actually infected the cells or is only attached to the cell surface. Therefore, this quantification approach essentially requires the virus to replicate to be detectable, i.e., amplification of the virus genome. We were able to detect an increased amount of virus in the cell pellet after an overnight incubation of about 17 h. This is in agreement with data previously published by Rosinski et al., stating the starting of DNA amplification 6 h post-infection and ending after about 20 h with the beginning of virus budding [42]. The focus of that earlier study was to characterize the kinetic of virus infection and to identify the relative percentage of virus budding from the cell after a high-titer infection. There, the infections were carried out at a fixed virus amount using an MOI of 20. Here, in this study, we wanted to quantify the number of infectious BVs by preparing a calibration curve with different virus concentrations up to an MOI of 1. The data suggest a reproducible DNA amplification in the infected cells over the whole calibration range (compare supplementary Figure S1). However, absolute quantification is difficult using this approach, as the initial virus amount is included in the data. Due to this, the differentiation between virus and nucleic acids bound to the outside of the cells without infecting them, virus that is infecting but not replicating, and actual replicating virus is not trivial. For future optimizations in that direction to enable a fast assessment of infectious virus particles, we suggest the detection of the viral (m)RNA that is transcribed in the cells by a qPCR or dPCR approach [43,44]. This, however, would involve a more sophisticated method development and could require the separation of viral DNA and (m)RNA to enable unambiguous results. With proper primer selection, the method could then be a fast alternative to estimate infectious virus titers. Simultaneously, it could be applied not only to various BV genotypes, e.g., by targeting (m)RNA of BV-gp64 protein, but would also be a feasible platform technology for different virus products, given the availability of suitable primers.

### 4.2. Flow Cytometric Quantification Based on Fluorophore Expression and Protein Labeling

We described the different detection possibilities using commonly applied flow cytometry approaches for BVs, which are either expressing GFP in infected cells or are native (wt). As was expected, the data show that only BV-GFP-infected cells can directly be detected to enable a successful quantification of the virus, whereas BV-wt requires an additional labeling procedure. The use of fluorophore-expressing viruses is common during process development on a research level for virus manufacturing and has been described for a variety of viruses [45,46,47]. However, viruses used for pharmaceutical applications lack the ability of fluorophore expression and require other means of detection and quantification. The same accounts for virus genotypes that encode for the expression of alternative marker proteins, which have previously been described [48]. For native or pharmaceutically relevant viruses, endpoint dilution assays are still the method of choice; however, these usually are cumbersome and inaccurate [11]. For comparison, the TCID_50_ and PFU assays we applied here to estimate the titer of the virus stock included a sample preparation and incubation time of 6–8 days. At the research level, different strategies have emerged to enable a faster and more reliable quantification. To name some examples, these include the detection of virus-related cell effects, e.g., increase in size [49], as well as the labeling of viruses (or virus-derived proteins) [23].

In order to to reduce the analysis time, allow in-process monitoring, and simultaneously enable the possibility of being used as a platform technology for various viruses, we evaluated the quantification based on a labeling of viral surface proteins in infected cells, in this case the BV-gp64 protein. Firstly, the data showed no differences in the results for similar virus concentrations of BV-wt as well as BV-GFP, indicating a robust and comparable performance irrespective of the virus genotype. Secondly, fluorescence signals for the labeled samples were already detectable within the first hours of incubation, whereas positive responses for the non-labeled BV-GFP could only be achieved after an overnight incubation. Accordingly, the optimum incubation time identified here for BV-GFP was between 16 and 18 h. Using this duration, the amount of fluorophore was high enough to allow unambiguous detection, and virus budding did not occur, thus avoiding secondary cell infections. Direct labeling of the baculoviral gp64 protein, on the other side, enables a faster detection, as the protein is part of the viral envelope of all BVs and is detectable on the cell surface of infected cells [50]. Thus, it is visible when measuring intact cells in the flow cytometer from the moment of virus attachment to the cell until cell lysis. The kinetic evaluation indicated, however, that the limit of detection as well as the reliability of the results, relating to standard deviation of replicates, increased with elongated incubation times, although these variations were lower for the APC-conjugated antibody, indicating a more sensitive and accurate detection. Accordingly, the optimum incubation times for PE and APC were considered to be at least 5 h and 4 h, respectively. The incubation times were chosen not only to allow a successful virus quantification but also to provide a robust and reproducible analysis. Accordingly, the APC procedure could, in principle, be evaluated after incubation times of less than one hour. However, detection requires at least successful virus attachment to the cells, which may vary between replicate measurements. Accordingly, at least several hours of infection are required to provide a statistically robust detection. Due to the higher sensitivity of the APC stain, the required incubation time is correspondingly shorter. The improved sensitivity of the APC was also confirmed during the optimization of the method, as lower antibody amounts, 0.0125 µg/sample compared to 0.05 µg/sample, and lower incubation times for the actual labeling, 15 min vs. 30 min, were required for the APC procedure (see Figure 3).

The upper limit of the linear calibration range for the three approaches was similar, with values in the range of 7 × 10^4^–2 × 10^5^ IU/mL. However, the limit of detection was the highest for the quantification based on GFP (1.5 × 10^4^ IU/mL) and was thus about 10 times higher than that for the labeling approaches, which had a limit of about 1 × 10^3^ IU/mL. Accordingly, the calibration ranges were narrower than for the qPCR evaluation, which allowed reliable quantification between 1.1 × 10^7^ IU/mL and 1.1 × 10^2^ IU/mL. Therefore, additional dilution steps are required prior to analysis for the flow cytometry approach when analyzing virus levels in process samples. Other calibration fits, such as logarithmic or exponential, could be used to increase the calibration range. However, these would require additional validation or verification efforts in order to enable a reproducible performance. We chose linear calibration to allow for simplified evaluation and to avoid secondary infections due to high virus concentrations. Additionally, it has to be mentioned that the fluorescence responses for GFP detection leveled off at about 35–40% positive cells, for virus concentrations above 1 × 10^6^ IU/mL, irrespective of an increase of the infectious virus amount to MOIs of 1 or 2. This could have several reasons, which shall be shortly discussed in the following. Firstly, the viral transduction efficiency might not be optimal, leading to lower expression rates of the GFP. Secondly, the fluorescent antibodies target the viral gp64 protein, which is present on the viral envelope and can thus be detected in the flow cytometer directly upon virus attachment to the cells. Although this gives an indication of a successful infection of the cells, it does not prove subsequent virus replication. Thus, depending on the activity of the virus, infectious titers might be overestimated. Nevertheless, the labeling of viral envelope proteins is a fast and feasible alternative to assess the virus amount in a process sample and is applicable as a platform technology for enveloped viruses. In the interest of completeness, it should be mentioned that all the described flow cytometric quantification approaches require a calibration standard of a known concentration. Therefore, the absolute quantification of this calibration standard has to be performed at least once by a classical endpoint dilution assay. However, for routine applications, it can greatly reduce the daily workload if this is not required for each sample.

### 4.3. Labeling of Virus-Derived mRNA in Infected Cells for Flow Cytometric Detection

As the above-mentioned methods are either not suitable to reliably quantify the amount of infectious viruses or can only be used as a platform technology for a certain class of viruses, we wanted to evaluate the possibility of a platform approach that is suitable for all kinds of viruses. The only target that is common to different virus classes and could allow early detection is the occurrence of viral mRNA during protein synthesis in the infected cells. Therefore, we performed a labeling of the mRNA of the BVs’ gp64 protein in infected cells as a proof of concept. Evaluations were started at MOI 1, as this would theoretically allow each cell to be infected by one virus, and thus was a promising basis for detecting positive signals. The data show that it is possible to detect the labeled mRNA with increasing responses within the first hours of infection. However, the standard deviation of replicate measurements was relatively high, and although the cells were infected at an MOI of 1, the signal-to-noise ratio was rather low. Accordingly, the (m)RNA appears to be a feasible target that can be detected in the infected cells prior to the corresponding proteins. However, several factors influence the success rate of labeling the virus-related RNAs and shall be shortly discussed in the following. Firstly, it was reported previously that six hours after the infection only about 3% of the overall mRNA levels in the infected cells is virus related and mainly caused by early promoting genes [51]. The percentage of viral mRNA increases then to about 38% after 12 h. The gp64 protein is encoded by genes that have both early and late promoters; hence, it is transcribed for a long time during the infection, with higher amounts available for elongated incubation times. Thus, by increasing the time before stopping the infection and starting the probe hybridization, the signal-to-noise ratio could be improved. Secondly, the number of viruses actually infecting the cells at a given time varies between different samples leading to increased standard deviation errors. Thus, the reliability of the data could either be improved by (again) increasing the incubation time of the infection and enhancing the probability of a rising mRNA content or by using a higher number of replicates. Thirdly, the RNA probe hybridization was not optimized for this proof of concept. This includes the accessibility of the target sequence inside the infected cell for the probe, as well as the probability of the probe reaching the respective counterpart. These issues may be improved by increasing the hybridization time (currently 2 h), enhancing the permeabilization procedure to open up cellular membranes, and finally by optimizing the target sequence of the probe to avoid a hindered attachment due to folded RNA regions. Once these aspects of the procedure have been optimized, an evaluation of various virus concentrations can be performed to determine a possible calibration range.

Nevertheless, several conclusions can be drawn from this initial proof of concept: The quantification of infectious viruses appears to be feasible by this approach, but optimum incubation times prior to analysis need to be identified and are likely to be between 5 h and 10 h. In addition, other aspects of the procedure, such as cell preparation, hybridization times, and probe composition will require extensive evaluation and improvement. Due to the time-consuming sample preparation for the mRNA labeling, the time to results is currently about 1–2 days, which is longer than the measurement of fluorophore expression (time to results less than 20 h) and longer than for the gp64 staining (less than 7 h), although the primary incubation times are similar to the latter. The current procedure involved a commercially available kit with costs per sample of about EUR 30, considering single measurements. Compared to the described qPCR approach (EUR 7/sample) and the labeling of viral envelope proteins (PE/APC: EUR 1/sample), these values are currently extraordinarily high.

## 5. Conclusions

When comparing the different analytical approaches described here for infectious BVs, each was found to have individual advantages over the others. While the qPCR approach results in rapid, accurate, and reproducible determination of viral titers based on the presence of viral DNA, the interference of an actual infectivity is not trivial. The cell culture-based assay presented here will require further optimization in the future to better distinguish between active and inactive viruses. The only real information on viral activity allowing a quantification was obtained either by fluorophore expression in infected cells using flow cytometric detection or by a traditional endpoint dilution approach with minimum analysis times of about 20 h and 7 d, respectively. For enveloped viruses, we described a rapid method based on the staining of viral surface proteins, which allows the quantification of virus titers as early as 4–5 h. However, this approach merely indicates the actual infectivity of the virus, as it also detects virus attached to the outer cell surface, which is not actively replicating. Finally, we presented a promising method to quantify the amount of infectious BVs based on viral (m)RNA labeling in infected cells. The method is based on in situ probe hybridization followed on by flow cytometric detection. Although the general detection principle is feasible according to the results of this study, rigorous future optimization will be required to improve the method’s performance and reduce the cost per analysis. Of the methods tested, this last approach has the potential to be developed into a platform analysis method for different types of viruses since viral (m)RNA is found in infected cells, irrespective of the production system, e.g., animal or insect cells, and irrespective of the virus composition, e.g., with and without envelope, or using RNA and DNA viruses. Thus, with substantial further improvements, this approach could greatly facilitate the process monitoring of viral vector and vaccine production at both research and manufacturing scales.

## Figures and Tables

**Table 1:** Optimum method parameters determined for the flow-cytometry-based quantification of BVs after the infection of Sf9 cells in a 96-well format. The procedure without staining is only suitable for viruses expressing a fluorophore (e.g., GFP), whereas the staining procedure targets the gp64 surface protein, which is present on all BVs, independent of the genotype.

Parameter [Unit]	No Staining	PE Staining	APC Staining
Seeding cell concentration [cells/mL]	0.8 × 106	1.0 × 106	1.0 × 106
Volume per well [µL]	150	150	150
Incubation time of virus infection [h]	17	5	4
Concentration of fluorescent antibody [mg/mL]	Not applicable	0.001	0.00025
Volume per well [µL]		50	50
Incubation time of antibody staining [min]	Not applicable	30	15 *

* The incubation can be performed at 4 °C or at room temperature, without affecting the results.

## Data Availability

The data presented in this study are available in the article. Further information is available on request from the corresponding author.

## Supplementary Materials

The following supporting information can be downloaded at https://www.mdpi.com/article/10.3390/v15040998/s1: Data S1: Additional data points for viral DNA quantification in the infected cell pellet. Figure S1: Amplification plots of the baculovirus DNA by qPCR measurements. The DNA was extracted from samples taken from the cell pellet of infected cells and amount of viral DNA detectable was monitored over the course of 7 h (A) and compared to values after 18 h of infection (B). For each incubation time, different virus concentrations at 10-fold dilutions (1.1 × 10^7^ IU/mL–1.1 × 10^3^ IU/mL) and a negative control were evaluated at each time point.
