# Supplementary material for: Evaluating Novel Quantification Methods for Infectious Baculoviruses
Source: Viruses. 2023 Apr 19;15(4):998. doi: 10.3390/v15040998 (PMC10141099; doi:10.3390/v15040998)
Supplement: Supplementary file 1 [file viruses-15-00998-s001.zip › viruses-2349162-supplementary.pdf]

# Supplementary Material:

## *Evaluating novel quantification methods for infectious baculoviruses*

### S1: Additional data points for viral DNA quantification in the infected cell pellet

The amount of viral DNA detectable in the cell pellet after the infection was monitored over the curse of 18 h (Supplementary figure 1). Each incubation time was analyzed with different virus concentrations ranging between 1.1E+07 IU/mL – 1.1E+03 IU/mL using 10-fold dilutions and a negative control (blank) sample. In the main text (Figure 2) only one virus concentration is depicted exemplarily in the interest of clarity to enable an easier observation of the data. However, similar trends were observed for the other applied concentrations as well and are depicted here for the complete overview.

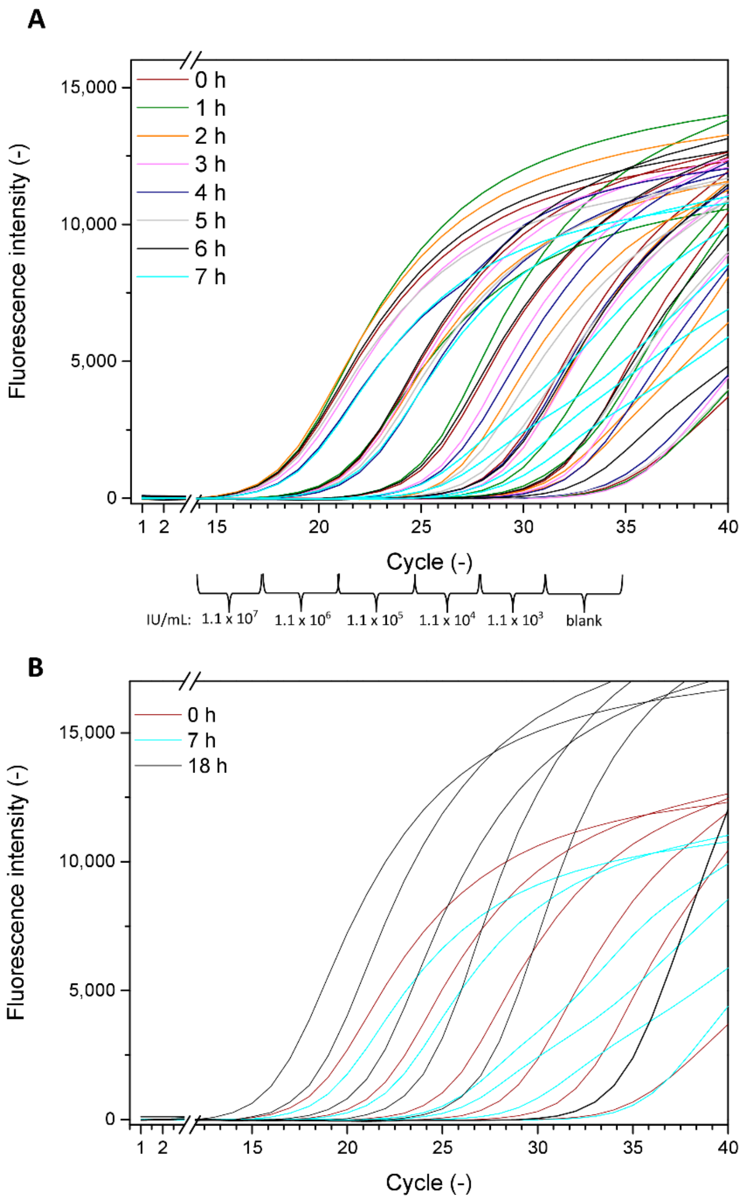

**Figure S1:** Amplification plots of the baculovirus DNA by qPCR measurements. The DNA was extracted from samples taken from the cell pellet of infected cells and amount of viral DNA detectable was monitored over the course of 7 h **(A)** and compared to values after 18 h of infection **(B)**. For each incubation time, different virus concentrations at 10-fold dilutions (1.1E+07 IU/mL – 1.1E+03 IU/mL) and a negative control were evaluated at each time point.
